# Supplementary material for: Comparative multiomics analysis of cell physiological state after culture in a basket bioreactor
Source: Sci Rep. 2022 Nov 23;12:20161. doi: 10.1038/s41598-022-24687-4 (PMC9686226; doi:10.1038/s41598-022-24687-4)
Supplement: Supplementary file 1 — Supplementary Information 1. [file 41598_2022_24687_MOESM1_ESM.zip › raw data/Transcriptomic raw data/6.Enrichment/1.GO/TE1vsTC1/GSEA/index.html]

Index for xtools.gsea.Gsea TE1vsTC1.Gsea.1656407547197


### GSEA Report for Dataset readcount

#### Enrichment in phenotype: **TE1 (2 samples)**

- 895 / 910 gene sets are upregulated in phenotype
   **TE1**
- 140 gene sets are significant at FDR < 25%
- 485 gene sets are significantly enriched at nominal pvalue < 1%
- 485 gene sets are significantly enriched at nominal pvalue < 5%
- Snapshot
  of enrichment results
- Detailed
  enrichment results in html
  format
- Detailed
  enrichment results in excel
  format (tab delimited text)
- Guide to
  interpret results

#### Enrichment in phenotype: **TC1 (2 samples)**

- 15 / 910 gene sets are upregulated in phenotype
   **TC1**
- 1 gene sets are significantly enriched at FDR < 25%
- 11 gene sets are significantly enriched at nominal pvalue < 1%
- 11 gene sets are significantly enriched at nominal pvalue < 5%
- Snapshot
  of enrichment results
- Detailed
  enrichment results in html
  format
- Detailed
  enrichment results in excel
  format (tab delimited text)
- Guide to
  interpret results

#### Dataset details

- The dataset has 22403 features (genes)
- No probe set => gene symbol collapsing was requested, so all 22403 features were used

#### Gene set details

- Gene set size filters (min=15, max=5000) resulted in filtering out 2487 / 3397 gene sets
- The remaining 910 gene sets were used in the analysis
- List of
  gene sets used and their sizes
  (restricted to features in the specified dataset)

#### Gene markers for the **TE1** *versus* **TC1** comparison

- The dataset has 22403 features (genes)
- # of markers for phenotype
   **TE1** 
  : 21031 (93.9% ) with correlation area NaN%
- # of markers for phenotype
   **TC1** 
  : 1372 (6.1% ) with correlation area 0.0%
- Detailed
  rank ordered gene list
  for all features in the dataset
- Heat map and gene list correlation
  profile for all features in the dataset
- Buttefly plot
  of significant genes

#### Global statistics and plots

- Plot of
  p-values
   *vs.* 
  NES
- Global ES
  histogram

#### Other

- Parameters
  used for this analysis

#### Comments

- Timestamp used as random seed: 1656407578868
- Warning: Phenotype permutation was performed but the number of samples in class A is < 7, phenotype: sample.cls
- Warning: Phenotype permutation was performed but the number of samples in class B is < 7, phenotype: sample.cls
- With small datasets, there might not be enough random permutations of sample labels to generate a sufficient null distribution. In such cases, gene\_set randomization might be a better choice.

---

Report: TE1vsTC1.Gsea.1656407547197.rpt   by user: liuyan6860

xtools.gsea.Gsea [Tue, Jun 28, '22 5 PM 12]

Website:
www.gsea-msigdb.org/gsea
Questions & Suggestions:
Contact page
